# Alanyl-Glutamine Dipeptide Mitigates *E. coli*-Induced Pathogenesis in *Hy-Line* Brown Hens: Mechanistic Insights into Microbiome Modulation, Antioxidant Response, and Intestinal Integrity

**DOI:** 10.3390/ijms27156936

**Published:** 2026-08-02

**Authors:** Usman Nazir, Zhi Yang, Muhammad Hammad Zafar, Xiaoli Wan, Haiming Yang

**Affiliations:** 1 College of Animal Science and Technology, Yangzhou University, Yangzhou 225009, China; 2 College of Bioscience and Biotechnology, Yangzhou University, Yangzhou 225009, China; 3 Joint International Research Laboratory of Agriculture and Agri-Product Safety of Ministry of Education of China, Yangzhou University, Yangzhou 225009, China

**Keywords:** *E. coli*, gut, diarrhea, challenge, antioxidant, microbiome, growing hens

## Abstract

*Escherichia coli* (*E. coli*) infection in poultry triggers severe diarrhea and intestinal damage, ultimately disrupting gut function. Alanyl-glutamine (Aln-Gln) dipeptide not only exhibits potent gut-repairing efficacy but also restores barrier integrity and counters *E. coli* pathology through mucosal healing and immune modulation. In this study, the effects of Aln-Gln on microbiome modulation and intestinal integrity in growing hens challenged with *E. coli* were evaluated. The 250 healthy, *Hy-line* brown pullets (42 days old) were randomly divided into 5 experimental groups, each having five replicates of 10 chicks. Five dietary groups include a basal diet without added Aln-Gln and no *E. coli* challenge (NC), a basal diet without added Aln-Gln and orally administered 2.0 × 10^4^ CFU/mL *E. coli* suspension (C), a basal diet added 0.1% Aln-Gln and orally administered 2.0 × 10^4^ CFU/mL *E. coli* suspension (G1), a basal diet added 0.2% Aln-Gln and orally administered 2.0 × 10^4^ CFU/mL *E. coli* suspension (G2) and a basal diet added 0.3% Aln-Gln and orally administered 2.0 × 10^4^ CFU/mL *E. coli* suspension (G3). The results showed that dietary supplementation with 0.2% or 0.3% Aln-Gln effectively mitigated the adverse effects of an *E. coli* challenge, restoring body weight and feed intake to levels comparable to non-challenged controls. Aln-Gln supplementation (0.2–0.3%) reduced diarrhea severity, restored ileal morphology (villus height, crypt depth), improved antioxidant enzyme activity (GSH-Px, SOD), and reduced oxidative markers (MDA) (*p* < 0.05). Aln-Gln decreased pro-inflammatory (IL-1β, IL-6, NO) and apoptotic (*BAX*) responses (*p* < 0.05) and enhanced barrier integrity. It rebalanced gut microbiota by suppressing pathogenic *Clostridium* (75% in C vs. 29.9% in G3) and increasing beneficial *Lactobacillus* in G2/G3 compared with C. These findings demonstrate Aln-Gln as a beneficial dietary additive that restores growth performance and gut integrity; reduces diarrhea severity, inflammation, and apoptosis; and improves antioxidant status as it rebalances gut microbiota.

## 1. Introduction

*Escherichia coli*-associated avian colibacillosis remains a pervasive challenge in poultry production, characterized by high morbidity, recurrent infections, and prolonged therapeutic interventions [1]. Unlike many infectious diseases, its incidence exhibits no significant correlation with seasonal variation, host age, or genetic breed, rendering it a ubiquitous threat to global poultry operations [2]. Complications arising from secondary infections further exacerbate economic losses, underscoring the urgency of developing alternative prophylactic strategies [3].

The gastrointestinal tract of growing hens undergoes critical developmental transitions during the post-hatch phase, marked by dynamic changes in intestinal epithelial structure, immune system maturation, and microbial colonization [4]. These physiological shifts coincide with heightened susceptibility to enteropathogens such as *E. coli*, particularly under conditions of dietary modification, husbandry-related stress, or incomplete establishment of commensal microbiota. Such disruptions to intestinal homeostasis often precipitate dysbiosis, compromising barrier function and creating niches for pathogenic invasion [5].

Glutamine (Gln), an occasionally essential amino acid, has emerged as a key modulator of intestinal health. As a primary energy substrate for enterocytes and lymphocytes, Gln supports epithelial repair, enhances tight junction protein expression (e.g., *occludin*, *ZO-1*), and mitigates oxidative stress through free radical scavenging [6]. Experimental evidence demonstrates its capacity to attenuate infection-induced apoptosis, restore mucosal integrity, and regulate immune responses via mast cell activation and cytokine modulation [7]. Furthermore, Gln supplementation has been shown to upregulate antimicrobial peptide synthesis, thereby enhancing innate defenses against enteric pathogens. However, free L-glutamine exhibits poor stability in aqueous solutions and is rapidly degraded during feed processing or in the gastrointestinal tract, limiting its bioavailability. Therefore, the Aln-Gln dipeptide serves as a superior alternative to glutamine monomers because of its significantly enhanced stability and solubility [8]. Interestingly, it accelerates intestinal villous growth and epithelial structure to enhance nutrient absorption, thereby improving growth and feed conversion efficiency.

The gut microbiota, dominated by Firmicutes, Bacteroidetes, and Proteobacteria at the phylum level, plays a pivotal role in maintaining intestinal equilibrium [9]. Key genera, including Lactobacillus, Clostridium, and Bacteroides, contribute to nutrient metabolism, competitive exclusion of pathogens, and immune system priming. Notably, Gln influences microbial community structure by promoting the proliferation of short-chain fatty acid (SCFA)-producing taxa while suppressing opportunistic pathogens, a dual mechanism that reinforces barrier function and immunological homeostasis [10,11].

This study examines the interplay between developmental gut physiology, *E. coli* pathogenicity, and Aln-Gln’s therapeutic potential. By synthesizing evidence on Aln-Gln’s roles in epithelial repair, immunomodulation, and microbiota regulation, we propose targeted dietary strategies to reduce reliance on broad-spectrum antibiotics in poultry management.

## 2. Results

### 2.1. Diarrhea Index

**Figure 1:**
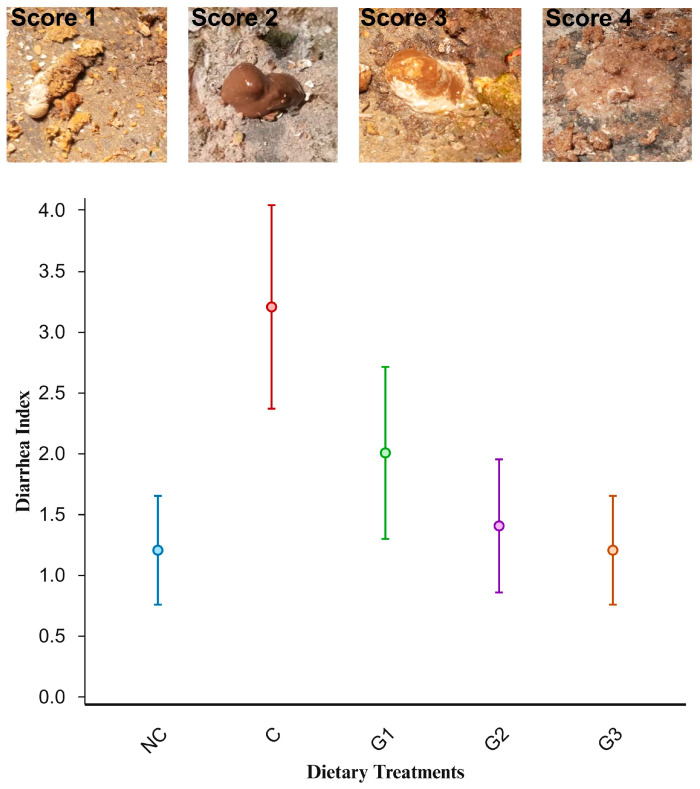
Diarrhea index in *E. coli*-infected growing hens. NC = Negative control group (no *E. coli* and no Aln-Gln); C = control group (*E. coli*-Challenged and no Aln-Gln); G1 = *E. coli*-Challenged and 0.1% Aln-Gln; G2 = *E. coli*-Challenged and 0.2% Aln-Gln; G3 = *E. coli*-Challenged and 0.3% Aln-Gln.

The effect of Aln-Gln on the diarrhea index of growing hens is shown in Figure 1. The *E. coli* challenge significantly increased the diarrhea index in group C (*p* < 0.001), confirming successful induction of diarrhea. Aln-Gln supplementation dose-dependently reduced diarrhea severity, with G2 and G3 demonstrating comparable efficacy to NC (*p* < 0.05).

### 2.2. Growth Performance

The *E. coli* challenge reduced (*p* < 0.05) final body weight (BW) and Average daily feed intake (ADFI) in hens (Table 1). Dietary supplementation with Aln-Gln at 0.2% and 0.3% mitigated these negative effects, restoring BW and ADFI to levels comparable to the NC group by d 56. Overall, the G1 treatment showed the most consistent and highest feed efficiency by day 56 (*p* < 0.05), while the NC group, despite increasing early on, dropped to the lowest efficiency (*p* < 0.05) by the end of the trial.

### 2.3. Ileal Morphology

The effect of different levels of Aln-Gln on ileal morphology of *Hy-line* brown growing hens is shown in Table 2. The villus length in the ileum was higher in groups NC, G2 and G3 than the other two groups (*p* < 0.05). However, crypt depth decreased in Aln-Gln supplemented groups and NC as compared to the control group. The villus-to-crypt ratio (V/C) was higher in NC, G2 and G3 than the control group (*p* < 0.05).

### 2.4. Serum Cytokine Content

The effects of dietary Aln-Gln levels on serum interleukin concentration in *E. coli*-Challenged growing hens are presented in Table 3. The *IL-1β*, *IL-6*, *IL-4*, and *IL-10* concentrations were significantly reduced in groups C and G1 compared to NC, G2, and G3 (*p* < 0.05), indicating that *E. coli* challenge suppressed anti-inflammatory responses, which were restored by 0.2–0.3%. Non-challenged groups showed lower TNF-α than all the tested and control groups (*p* < 0.05).

### 2.5. Serum Antioxidant Status

The effects of dietary Aln-Gln on the serum antioxidant status of growing hens are shown in Table 4. Group C significantly reduced serum GSH-Px, CAT, and SOD activities while elevating MDA levels compared to the NC (*p* < 0.05). Aln-Gln supplementation restored antioxidant enzyme activities and reduced MDA (*p* < 0.05), with 0.2–0.3% Aln-Gln showing normalization of oxidative stress markers. These results indicate that Aln-Gln mitigates *E. coli*-induced oxidative damage in a dose-responsive manner.

### 2.6. Intestinal Mucosal NO and NOS Levels

The effects of Aln-Gln supplementation on the nitric oxide (NO) and nitric oxide synthase (NOS) levels in the ileum mucosae of growing hens infected with *E. coli* are shown in Table 5. Dietary Aln-Gln supplementation decreased NO activity in ileal mucosae of growing hens as compared to group C (*p* < 0.05). The inducible NO synthase (iNOS) and total nitric oxide synthase (TNOS) activities in ileal mucosa were increased in group C and G1 as compared to NC and other dietary groups (*p* < 0.05).

### 2.7. Composition of Gut Flora and Significance Analyses of Differences Between Groups

**Figure 2:**
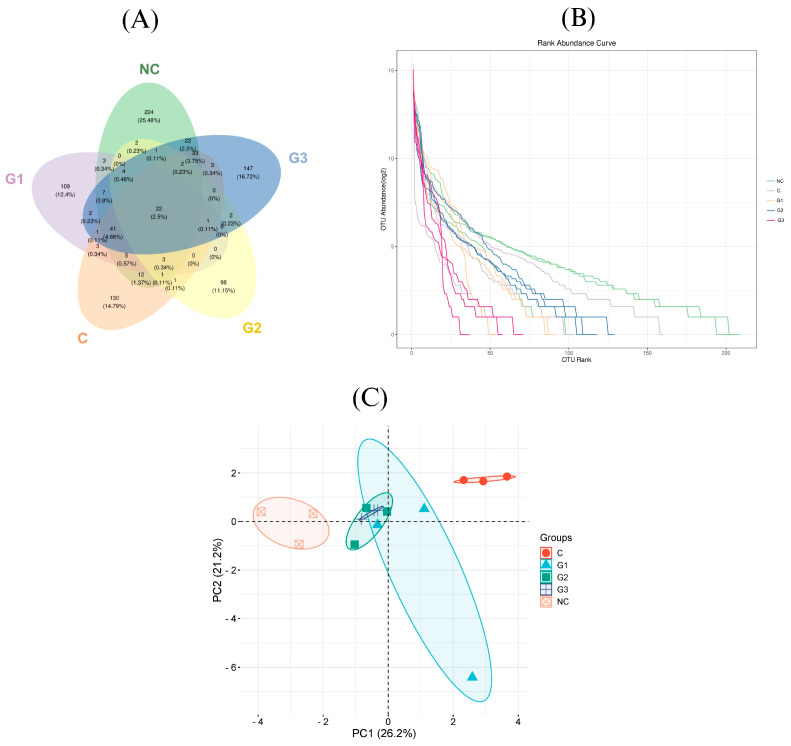
16S rRNA analysis of the classification and composition of gut microbiota in chicken. (**A**) Venn diagram of growing hen microorganisms; (**B**) OUT abundance among groups; (**C**) PCoA plot showing beta diversity; NC = negative control group (no *E. coli* and no Aln-Gln); C = control group (*E. coli*-Challenged and no Aln-Gln); G1 = *E. coli*-Challenged and 0.1% Aln-Gln; G2 = *E. coli*-Challenged and 0.2% Aln-Gln; G3 = *E. coli*-Challenged and 0.3% Aln-Gln.

Analysis of the noise-filtered sequencing data revealed 881 Amplicon Sequence Variants (ASVs) with distinct group-specific distributions: 224 features were unique to the NC group, 130 to C, 109 to G1, 98 to G2, and 147 to G3, while 173 features were shared across all groups, highlighting a conserved core microbial community (Figure 2A).

**Figure 3:**
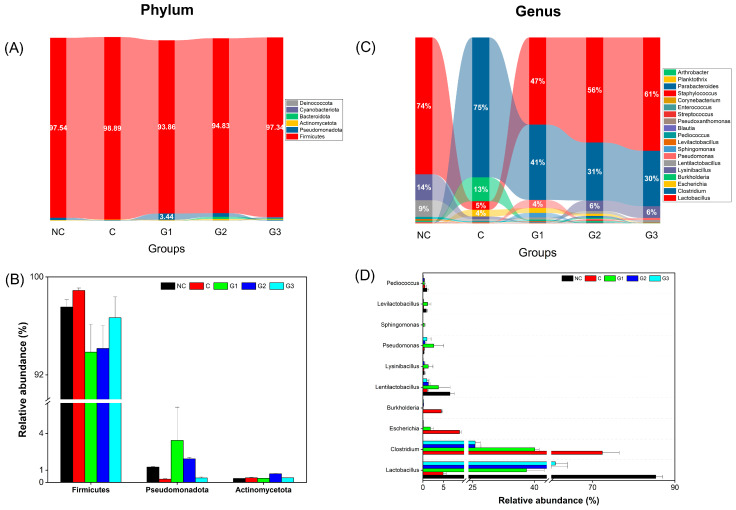
16S rRNA analysis of the classification and composition of gut microbiota in chicken. (**A**,**B**) Composition and differences in gut microbiota at the phylum level; (**C**,**D**) composition and differences in gut microbiota at the genus level; NC = negative control group (no *E. coli* and no Aln-Gln); C = control group (*E. coli*-Challenged and no Aln-Gln); G1 = *E. coli*-Challenged and 0.1% Aln-Gln; G2 = *E. coli*-Challenged and 0.2% Aln-Gln; G3 = *E. coli*-Challenged and 0.3% Aln-Gln.

At the phylum level, *Firmicutes* dominated all experimental groups, constituting 97.54% (NC), 98.89% (C), 93.86% (G1), 94.83% (G2), and 97.34% (G3) of microbial composition. Genus-level profiling uncovered significant shifts between groups: *Clostridium* and *Burkholderia* were predominant in the C group (75% and 13%, respectively), with decreasing abundances in G1 (41% and 1.59%), G2 (31% and 1.1%), and G3 (29.90% and 0.93%). In contrast, the NC group was characterized by high abundances of *Lactobacillus* (74%), *Lysinibacillus* (14%), and *Lentilactobacillus* (9%). The C group, however, showed minimal representation of these taxa, with *Escherichia* (4%) and *Lactobacillus* (5%) emerging as minor constituents (Figure 3). Together, these findings underscore distinct microbial signatures across groups, with *Firmicutes* as the phylum-level cornerstone and dynamic genus-level variations reflecting experimental conditions.

### 2.8. Alpha Diversity Analysis

**Figure 4:**
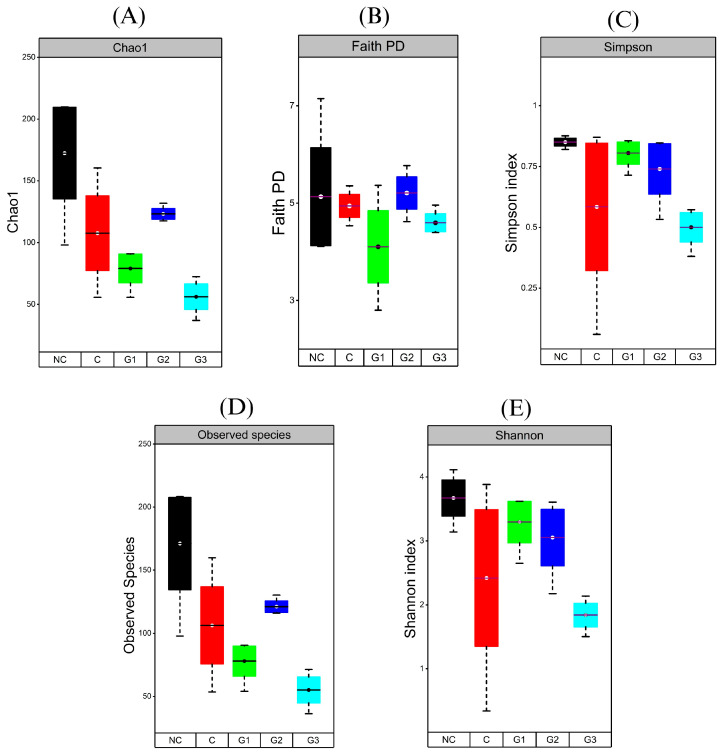
Alpha diversity of chicken gut microbiota. (**A**) Chao1 index; (**B**) Faith PD index; (**C**) Simpson index; (**D**) Observed Species index; (**E**) Shannon index; NC = negative control group (no *E. coli* and no Aln-Gln); C = control group (*E. coli*-Challenged and no Aln-Gln); G1 = *E. coli*-Challenged and 0.1% Aln-Gln; G2 = *E. coli*-Challenged and 0.2% Aln-Gln; G3 = *E. coli*-Challenged and 0.3% Aln-Gln.

Alpha diversity analysis across treatments revealed distinct patterns in microbial community richness and evenness (Figure 4). The Chao1 index and Observed Species metrics indicated the highest species richness in the NC group, followed by a decline in C and progressively lower values in G1, G2, and G3. Faith PD (phylogenetic diversity) also showed a similar trend, with the NC group showing the greatest evolutionary divergence, while G1–G3 displayed reduced diversity. The Shannon index, reflecting species evenness and richness, was highest in NC, moderately lower in C, and decreased incrementally across G1–G3. Conversely, the Simpson index, emphasizing dominance, suggested reduced evenness in G1–G3 compared to NC and C. These results highlight a gradient of declining alpha diversity from the NC group through G1–G3, with C occupying an intermediate position, underscoring group-specific impacts on microbial community structure.

### 2.9. Beta Diversity Analysis

The PC plot, based on beta diversity, revealed distinct clustering patterns among the experimental groups. The first two principal coordinates (PC1: 26.2%, PC2: 21.2%) explained a cumulative 47.4% of the total variance. Groups G1, G2, and G3 exhibited closer proximity to one another along the ordination axes, suggesting shared microbial community structures. In contrast, the NC group and C group occupied distinct regions of the plot, indicating divergent community compositions. Statistical validation via ANOSIM analysis confirmed significant differences between groups (R = 0.392, *p* < 0.001), underscoring the reliability of these separations. These results highlight a gradient of microbial dissimilarity with G1–G3 forming a cohesive cluster distinct from C and NC, reflecting experimental impacts on community dynamics (Figure 2C).

### 2.10. KEGG Enrichment Analysis for Identification of Abundant Metabolic Pathways

**Figure 5:**
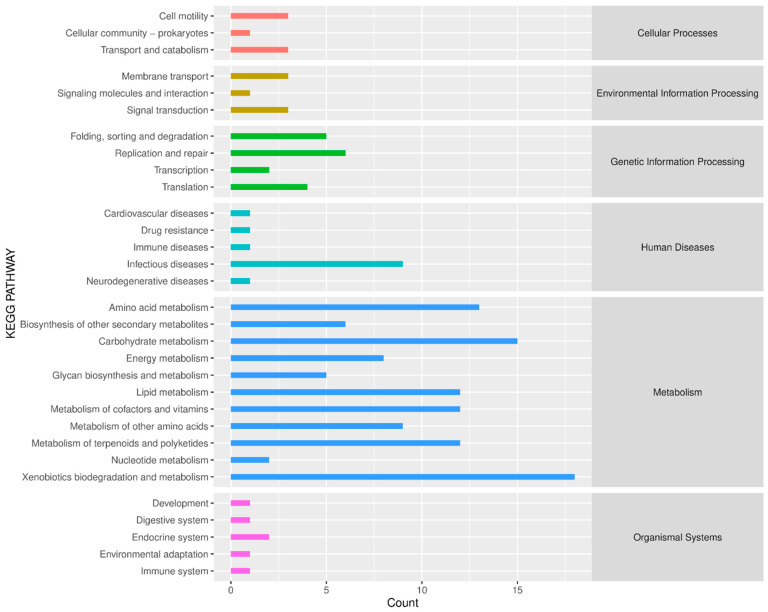
Enrichment analysis for different metabolic pathways.

Subsequent KEGG enrichment analysis revealed significant pathway alterations underpinning Ala-Gln’s protective mechanisms in *E. coli*-Challenged hens. Key enriched pathways were hierarchically clustered within three major functional categories: (1) metabolism, particularly amino acid, carbohydrate, and lipid metabolism reflecting enhanced nutrient utilization for energy homeostasis and mucosal repair; (2) organismal systems, notably digestive and immune system pathways directly supporting gut barrier restoration and inflammatory response modulation; and (3) diseases, including infectious diseases and drug resistance pathways demonstrating enhanced pathogen defense. This coordinated enrichment across fundamental metabolic, physiological, and disease-resistance mechanisms provides systematic validation of Ala-Gln’s role in optimizing intestinal health during enteric challenges through microbiome modulation, antioxidant activity, and epithelial integrity preservation (Figure 5).

### 2.11. Relative mRNA Expression of Genes

**Figure 6:**
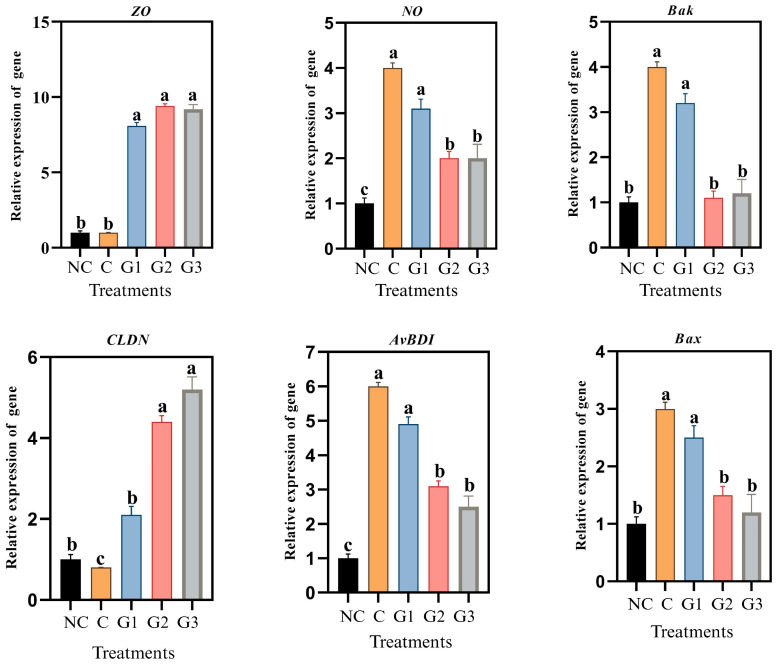
Relative gene expression results by qRT-PCR, presented as mean ± SEM. NC = Negative control group (no *E. coli* and no Aln-Gln); C = control group (*E. coli*-Challenged and no Aln-Gln); G1 = *E. coli*-Challenged and 0.1% Aln-Gln; G2 = *E. coli*-Challenged and 0.2% Aln-Gln; G3 = *E. coli*-Challenged and 0.3% Aln-Gln. Superscripts in the same row indicate significant differences at *p* < 0.05.

The relative expression of *AvBDI*, *Bak*, *NO*, and *BAX* increased in the C group compared to NC, showing enhanced immune and apoptotic responses to infection (*p* < 0.05). Aln-Gln supplementation showed a dose-dependent attenuation of these effects, with G2 and G3 normalizing *AvBDI*, *Bak*, and *BAX* expression toward NC levels, suggesting protective modulation of immune and apoptotic pathways. Conversely, *CLDN* and *ZO-1* expressions were reduced in the C group but progressively restored in G1–G3 (*p* < 0.05), indicating Aln-Gln’s role in enhancing gut barrier integrity (Figure 6).

## 3. Discussion

The findings of this study collectively demonstrate that dietary supplementation with Aln-Gln effectively mitigates *Escherichia coli*-induced diarrhea in growing hens. This amelioration is achieved by restoring gut integrity, modulating local immune and oxidative stress responses, and beneficially reshaping the gut microbial ecology. The resulting improvements in nutrient absorption and host health directly translated to the observed recovery of body weight and feed intake following the challenge. These results align with previous studies and confirm Aln-Gln’s role as a potent functional nutrient for supporting gut health in poultry production [1,12,13]. The severe diarrhea observed in the *E. coli*-Challenged control group (C) aligns with the pathogen’s known disruption of intestinal barrier function, as evidenced by improved gut health and reduced villus-to-crypt (V:C) ratios. These morphological distortions impair nutrient absorption and increase intestinal permeability, facilitating fluid leakage and diarrhea [14]. However, Aln-Gln supplementation reversed these effects, significantly elevating villus height, normalizing crypt depth, and improving V:C ratios. This restoration of ileal architecture underscores glutamine’s critical role as an energy source for enterocytes, driving mucosal repair and epithelial regeneration [15,16]. Meanwhile, the upregulation of tight junction proteins in Aln-Gln groups further improved gut barrier integrity, reducing permeability and physically limiting *E. coli* translocation, a key factor in resolving diarrhea and preventing systemic complications like pericarditis [17].

The anti-inflammatory properties of Aln-Gln were evident in its modulation of serum cytokines [18]. The elevated pro-inflammatory markers (IL-1β, IL-6, TNF-α) in Group C reflect a hyperactive immune response to *E. coli* lipopolysaccharides (LPS), which intensifies mucosal damage and systemic inflammation [19]. Aln-Gln’s suppression of these cytokines, particularly in higher-dose groups (G2, G3), highlights its ability to reduce NF-κB signaling, a central pathway in inflammatory cascades [15]. Ironically, the transient rise in anti-inflammatory cytokines (IL-4, IL-10) in Group C and low-dose Aln-Gln (G1) suggests a compensatory feedback mechanism to counterbalance inflammation [7,20]. The elevated pro-inflammatory markers (IL-1β, IL-6, TNF-α) in Group C reflect a hyperactive immune response to *E. coli* lipopolysaccharides (LPS), which intensifies mucosal damage and systemic inflammation [18]. Aln-Gln’s suppression of these cytokines, particularly in higher-dose groups (G2, G3), highlights its ability to reduce NF-κB signaling, a central pathway in inflammatory cascades [14]. Concurrently, the restoration of anti-inflammatory cytokines IL-4 and IL-10 in G2/G3 suggests that adequate Aln-Gln doses promote immune homeostasis, shifting the balance from a pro-inflammatory toward a regulated response [21]. This dual modulation of pro- and anti-inflammatory signals underscores Aln-Gln’s capacity to restore immune homeostasis, curtailing both local and systemic pathology [22].

Oxidative stress, a marker of *E. coli* infection, was significantly alleviated by Aln-Gln. The depletion of antioxidant enzymes (GSH-Px, CAT, SOD) and elevated MDA in Group C indicate widespread lipid peroxidation and cellular damage [12,22]. Gln’s dose-dependent improvement in antioxidant capacity likely stems from its role as a precursor for glutathione synthesis and its direct scavenging of reactive oxygen species [13]. The reduction in ileal NO and iNOS activity further highlights its antioxidant efficacy, as excessive NO production during infection generates cytotoxic peroxynitrite, which leads to increased mucosal injury [23]. By neutralizing oxidative stress, Gln preserves enterocyte viability, synergizing with its anti-inflammatory effects to maintain gut barrier function [4].

Microbial community analysis revealed profound shifts in gut microbiota composition driven by *E. coli* challenge and Aln-Gln intervention. While *Firmicutes* dominated all groups, the proliferation of pathobionts like *Clostridium* and *Burkholderia* in Group C underscores the dysbiosis typical of enteric infections. These genera are linked to inflammation [24] and opportunistic pathogenesis causing mucosal damage [25]. In contrast, Gln supplementation enriched beneficial taxa, such as *Lactobacillus*, a genus renowned for its anti-pathogenic and immunomodulatory properties [26]. The decline in alpha diversity (Chao1 and Shannon indices) in Aln-Gln groups may initially seem contradictory, but likely reflects a targeted suppression of pathogens (*Escherichia*, *Clostridium*) and enrichment of symbionts, yielding a functionally optimized but less diverse microbiota [27]. Beta diversity further supports this change, with G1–G3 clustering separately from C and NC, indicative of Gln’s microbial-modulating efficacy [28]. The conserved core ASVs across groups suggest that Gln selectively reshapes microbiota without eradicating foundational communities, preserving ecological stability [29].

The dietary inclusion of Aln-Gln induced significant microbial restructuring in *E. coli*-Challenged hens, characterized by a dramatic reduction in pathogenic *Clostridium*. These changes align with amino acid metabolism pathways [30], where Aln-Gln, as a glutamine-containing dipeptide, likely serves as a critical substrate for enterocytes and the microbiota. Enhanced glutamine utilization may fuel mucosal repair (evidenced by improved villus height and crypt depth) while providing metabolic precursors for *Lactobacillus* proliferation, which thrives on amino acid-derived fermentation products [31,32,33]. This microbial shift further correlates with carbohydrate metabolism pathways, as *Lactobacillus* generates short-chain fatty acids (SCFAs) that inhibit *E. coli* colonization and fortify mucosal immunity. Simultaneously, the decline in *Clostridium* aligns with xenobiotics biodegradation and drug resistance pathways, suggesting Aln-Gln enhances detoxification processes (e.g., glutathione synthesis) to neutralize bacterial toxins, thereby mitigating oxidative stress (↓*MDA*, ↑*GSH-Px/SOD*) and inflammation [34,35,36]. The interplay of amino acid metabolism and microbial rebalancing also supports membrane transport pathways, improving nutrient absorption and tight junction integrity, which synergizes with Aln-Gln’s barrier-restoring effects [37].

At the molecular level, Aln-Gln’s normalization of pro-apoptotic genes (Bak, BAX) and antimicrobial peptide AvBD1 in challenged birds highlights its role in balancing epithelial turnover and immune defense. The upregulation of these markers in Group C reveals the host’s attempt to eliminate infected cells and pathogens [38], while excessive apoptosis aggravates epithelial loss, perpetuating diarrhea [39]. Aln-Gln’s dose-dependent attenuation of apoptotic signaling preserves epithelial continuity, while the restoration of tight junction proteins (CLDN, ZO-1) strengthens the physical barrier against pathogen invasion. The reduction in AvBD1 expression in treated groups further suggests that enhanced barrier function and microbiota modulation reduced dependence on defending mediated antimicrobial activity, showing Aln-Gln’s multifaceted protective mechanisms [23].

Aln-Gln’s ability to synchronize gut morphology, immune responses, oxidative balance and microbial ecology makes it a key component for sustainable poultry health management. By targeting multiple facets of *E. coli* pathogenesis, it not only resolves diarrhea but also mitigates systemic complications, offering a scientifically robust solution to one of the poultry industry’s most pressing challenges.

## 4. Materials and Methods

### 4.1. Animal Care

The current experiment and protocols were performed following the YZU Animal Care and Use Committee of Yangzhou University, Jiangsu, China (under permission number SYXK [Su] 2021-0020).

### 4.2. Preparation of the E. coli Inoculum

A multidrug-resistant, virulent *E. coli* isolate recovered from diseased poultry birds was used in this study [40]. A total of 100 µL of bacterial culture was taken from a frozen stock aliquot and added to a 1 mL Eppendorf tube containing tryptic soy broth (TSB) (Difco, Detroit, MI, USA) and subjected to overnight incubation at 37 °C. Next, a loop full of overnight-cultured bacterial culture was streaked on MacConkey agar (Oxoid, Basingstoke, UK), and plates were incubated for 16–18 h at 37 °C. Following incubation, an isolated pink colony from a MacConkey agar plate was picked using a sterile swab and suspended again in 1 mL TSB and incubated for 6 h with shaking at a speed of 200 rpm to get the log phase of bacterial culture. Cultured *E. coli* was deliquated to 2.0 × 10^4^ CFU/mL in a sterile physiological salt mixture to make a suspension.

### 4.3. Experimental Diets and Analysis

The test diets were based on soyabean and corn with Aln-Gln added at 0%, 0.1%, 0.2%, and 0.3%. They were formulated (Table 6) to meet the nutritional requirements for Hy-line brown growing hens according to the Hy-Line Management Guide [41] and validated against NRC [42]. A powdered form of Aln-Gln (Shandong Chen-long Pharmaceutical Co., Ltd., Jining, China; purity > 99%) was precisely weighed and mixed with the basal diet using a stepwise dilution method. Initially, Aln-Gln was blended with a small portion of corn (1 kg) in a laboratory mixer for 10 min, followed by incorporation into the complete diet using a horizontal ribbon mixer (SLHSJ-0.2, Jiangsu Muyang Group Co., Ltd., Yangzhou, China) for 15 min to ensure uniform distribution. In accordance with the methods of the Association of Official Analytical Chemists [43], the diets were analyzed for crude protein (method 990.03) using the automatic Kjeldahl nitrogen analyzer (Kjeldahl Nitrogen Analyzer Kd520, Jinan Alva Instrument Co., Ltd., Jinan, China). Sodium (Na) concentration was determined using flame photometry (AOAC method 984.27) after wet acid digestion, while chloride (Cl) content was quantified via silver nitrate titration using the Mohr method (AOAC method 971.27). Available phosphorus (non-phytate phosphorus) was not directly measured but calculated based on the ingredient-specific phytate:total phosphorus ratios. Total phosphorus was analyzed via UV-Vis spectrophotometry (AOAC method 985.01), and phytate phosphorus was determined using anion-exchange chromatography (AOAC method 986.15). Available phosphorus was subsequently derived as the difference between total phosphorus and phytate-bound phosphorus, in accordance with standard NRC [42] bioavailability coefficients for corn-soybean meal-based diets. Crude fiber was analyzed according to AOAC Official Method 962.09. Calcium and total phosphorus (method 985.01) were assayed using the acid-burette (50 mL, Tianjin Tianke Glass Instrument Manufacturing Co., Ltd., Tianjin, China) and the UV-Vis spectrophotometer (Thermo Scientific Multiskan FC, Thermo Fisher Scientific Inc. Co., Ltd., Shanghai, China). Amino acids (method 982.30), including methionine and lysine, were quantified using an automatic amino acid analyzer (L-8900, Hitachi Ltd., Tokyo, Japan). The amino acid values are reported as standardized ileal digestibility, which was determined by collecting terminal ileal digesta after a 5-day adaptation period and correcting apparent ileal digestibility for basal endogenous amino acid losses obtained from birds fed a nitrogen-free diet. Additionally, to verify the graded increment of the Aln-Gln dipeptide in the experimental diets, the constituent amino acids (alanine and glutamine) were also quantified using the same automatic amino acid analyzer, which confirmed that their dietary concentrations increased proportionally with the graded inclusion levels of Aln-Gln (0%, 0.1%, 0.2%, and 0.3%). Gross energy of feed samples was determined using a bomb calorimeter (HYLRY 6000, Hebi Huayuan Instrument & Meter Co., Ltd., Hebi, China), and metabolizable energy was calculated accordingly. Organic matter content was determined by subtracting crude ash (determined using a Muffle Furnace, SX2-4-10N, Shanghai Yiheng Scientific Instruments Co., Ltd., Shanghai, China) from dry matter.

### 4.4. Experimental Design

A total of 250 healthy, 42-day-old *Hy-line* Brown growing hens with similar body weight (438 ± 5.2 g) were randomly allocated into 5 experimental groups (n = 50 per group), each comprising 5 replicates of 10 birds. Randomization was performed using a computer-generated random number table (Microsoft Excel, RAND function). Birds were stratified by initial body weight to ensure uniform distribution across groups, and replicates within each group were balanced for cage position to minimize environmental variation. All chicks were raised according to the Management Guide for Hy-Line Brown Hens in bird cages measuring 37 × 30 × 40 cm, equipped with external access devices that constantly supplied water and feed throughout the experiment. The five groups were designed as (1) a basal diet (NC group; n = 50) without Aln-Gln and no *E. coli* challenge; (2) a basal diet without added Aln-Gln and orally administered 2.0 × 10^4^ CFU/mL *E. coli* suspension (C group, 2.0 mL per bird; n = 50); (3) a basal diet with 0.1% Aln-Gln and orally administered 2.0 × 10^4^ CFU/mL *E. coli* suspension (G1 group, 2.0 mL per bird; n = 50); (4) a basal diet with 0.2% Aln-Gln and orally administered 2.0 × 10^4^ CFU/mL *E. coli* suspension (G2 group, 2.0 mL per bird; n = 50); (5) a basal diet with 0.3% Aln-Gln and orally administered 2.0 × 10^4^ CFU/mL *E. coli* suspension (G3 group, 2.0 mL per bird; n = 50). Each bird in the C, G1, G2, and G3 groups was orally administered 2.0 mL of *E. coli* suspension on experimental day 3. Body weight and feeding intake were measured on a per-bird and per-pen basis, respectively, at weekly intervals throughout the trial.

### 4.5. Diarrhea Index

On experimental day 14, fecal samples were examined in the cage trays of birds. Diarrhea index was assessed in each replicate using a standardized ordinal scoring system.

1. Score 1: No diarrhea; solid fecal matter.
2. Score 2: Mild diarrhea; soft fecal matter.
3. Score 3: Moderate diarrhea; loose fecal matter.
4. Score 4: Severe diarrhea; watery fecal matter.

Observations were conducted by random evaluators to minimize bias, with scores aggregated per experimental treatment for statistical analysis.

### 4.6. Tissue Sample Collection

On the 14th day of the experiment, following the approved procedures of the YZU Animal Care Advisory Committee, one bird of average body weight from each replicate was selected and slaughtered using the knife method. From the small intestine, the ileum section was separated, washed, and fixed in 4% paraformaldehyde solution for anatomical study. By rubbing the microscopic slide on ileal fragments, the mucosal samples were collected and then stored at −80 °C for further microbiome and chemical studies.

### 4.7. Ileal Morphological Analysis

The ileal segments were embedded in paraffin. The 5 µm thick tissue sections were subjected to de-paraffinization, rehydration, and staining with hematoxylin and eosin. A minimum of three films per fragment and three segments from each bird were acquired for analysis. After the collection of ileal mucosal samples, the villus height (VH) and crypt depth (CD) were measured using an NIKON YS100 light microscope (Nikon Instruments Inc. Tokyo, Japan). The average measurements were then used to calculate the V:C.

### 4.8. Growth Performance

Feed intake and body weight of all the replicates were recorded on a weekly basis, while phase-wise calculation of body weight gain (BWG), average daily feed intake (ADFI), and feed conversion efficiency (FCE) was done. Phase selection was based on observing the impact of rearing period on growth performance. Moreover, the above parameters were also corrected for mortality.

### 4.9. Mucosal and Serum Indices

After 12 h of fasting on day 14 of the experiment, 2 birds were arbitrarily selected from each replicate (10 birds per group). The blood samples of each selected bird were collected using vacutainers with a clot activator. Afterward, these tubes were placed in a water bath (37 °C) for 10 min and then centrifuged at 3000 rpm for 10 min to separate serum. The collected serum samples were preserved at −20 °C for analysis of interleukin-1 (IL-1), interleukin-4 (IL-4), interleukin-6 (IL-6), and interleukin-10 (IL-10) by using ELISA kits from Nanjing Jiancheng Bioengineering Institute (Nanjing, China). The collected gut tissue samples were used for superoxide dismutase activity (SOD, cat. No. A001-2), catalase (CAT, cat. No. A007-1-1), malondialdehyde (MDA, cat. No. A003-1-2), and glutathione peroxidase activity (GSH-Px, cat. No. A005-1-1) by following the procedures of Jazideh et al. [44] method, using commercial kits according to manufacturer instructions (Nanjing Jiancheng Bioengineering Institute, Nanjing, China).

Ileum mucosae were homogenized, centrifuged, and then the supernatant was collected for analyses of NO and NOS levels. The activities of NO and NOS in the samples were assayed using commercial assay kits (Jiancheng Bioengineering Institute, Nanjing, China).

### 4.10. Gene Expression Analysis by qRT-PCR

Quantitative real-time PCR (qRT-PCR) was conducted to determine the relative mRNA expression of selected genes involved in apoptosis (*BAX*, *Bak*), tight junction integrity (CLDN, ZO-1), immune defense (AvBD1), and nitric oxide synthesis (NOS2). The TRIzol reagent (Invitrogen, Carlsbad, CA, USA) was used to isolate total RNA from ileal mucosal samples (n = 5 per treatment, one bird per replicate). The concentration of isolated RNA was measured using NanoDrop2000 (Thermo Fisher Scientific). The 1 mg of RNA was used to prepare reverse transcription with the Prime Script-RT reagent kit and gDNA Eraser. The amplification was performed using TB Green Premix Ex Taq II (Takara Bio SYBR Green, San Jose, CA, USA) on a Bio-Rad CFX384 real-time PCR system (Bio-Rad Laboratories, Inc., Hercules, CA, USA). The expression levels were measured using the 2^ΔΔCt^ method, with β-actin serving as the control, following the method of Kim et al. [45]. The primer sequences for the genes are presented in Table 7. All primers were designed by AZENTA Life Sciences Co., Ltd., located in Waltham, MA, USA.

### 4.11. 16s rRNA Sequence Analysis of Gut Contents

Ileum samples were collected, and total DNA extraction was done with the TGuide S96 fecal DNA kit (Tiangen Biochemical Technology (Beijing) Co., Ltd., Beijing, China) according to the instructions mentioned by the company. The concentration and purification of DNA were calculated through a NanoDrop 2000 UV-Vis spectrophotometer (Thermo Scientific, Wilmington, NC, USA). The quality of the DNA was checked through 1.8% agarose gel electrophoresis. The V3-V4 hypervariable regions of the bacterial 16S rRNA gene were amplified with primers 338F: 5′-ACTCCTACGGGAGGCAGCA-3′ and 806R: 5′-TAAT-3′ in a standard PCR system. The amplified products were again purified using the Omega DNA Purification Kit (Omega Inc., Norcross, GA, USA). The purified PCR products were collected and sequenced in pairs (2 × 250 bp) on the Illumina Novaseq 6000 platform (Illumina, Inc., San Diego, CA, USA). The raw data were filtered using Trimmatic (version 0.33) and UCHIME (version 8.1) to produce high-quality reads and amplicon sequence variants (ASVs). These were clustered/de-noised and separated into OTUs/ASVs. The species classification was determined according to the sequence composition of the features, with taxonomic analysis carried out at each taxonomic level. Alpha and Beta diversity analyses were employed to assess species diversity within each individual sample and to identify differences in species diversity (community composition and structure) between samples. Only those bacterial taxa with an abundance > 0.1% in at least one sample were analyzed. Statistically significant differences were observed among the different groups. Functional profiles of microbial communities were predicted from 16S rRNA gene sequencing data using PICRUSt2 (Phylogenetic Investigation of Communities by Reconstruction of Unobserved States, version 2.3.0-b). Predicted metagenomes were annotated against the KEGG database (Kyoto Encyclopedia of Genes and Genomes, Release 99.0), and pathway enrichment analysis was performed using the STAMP software package (version 2.1.3) with Fisher’s exact test and Benjamini–Hochberg FDR correction (*p* < 0.05).

### 4.12. Statistical Analysis

All data were analyzed using SPSS version 17.0 (SPSS Inc., Chicago, IL, USA). Normality of residuals and homogeneity of variances were tested using the Shapiro–Wilk test and Levene’s test, respectively. Data that met these assumptions were analyzed by one-way analysis of variance (ANOVA) using the following general linear model:

Y_ij = μ + α_i + ε_ij

where Y_ij is the dependent variable, μ is the overall population mean, α_i is the fixed effect of the i-th dietary treatment (i = NC, C, G1, G2, G3), and ε_ij is the random experimental error term, assumed to be independently and normally distributed with mean zero and variance σ^2^.

For growth performance parameters (BW, ADFI), the replicate pen (n = 5 per treatment) was considered the experimental unit. For all other parameters (serum cytokines, antioxidant enzymes, gene expression, etc.), individual birds (n = 5 per treatment, one per replicate) were considered the experimental units. When significant treatment effects were detected (*p* < 0.05), means were compared using Fisher’s Least Significant Difference (LSD) post hoc test.

For microbiota data, alpha diversity indices (Chao1, Observed Species, Faith PD, Shannon, Simpson) were compared among groups using the Kruskal–Wallis test followed by Dunn’s post hoc test with Benjamini–Hochberg false discovery rate (FDR) correction. Beta diversity was assessed using principal coordinate analysis (PCoA) based on Bray–Curtis dissimilarity matrices, with statistical significance tested by permutational multivariate analysis of variance (PERMANOVA, adonis function with 999 permutations). Linear discriminant analysis effect size (LEfSe) was performed to identify differentially abundant taxa among groups (threshold: LDA score > 2.0, *p* < 0.05). All figures were generated using GraphPad Prism version 8.0 (GraphPad Software Inc., San Diego, CA, USA).

## 5. Conclusions

Dietary Aln-Gln effectively mitigates *E. coli*-induced diarrhea in poultry by restoring gut integrity through enhanced villus morphology, tightened epithelial junctions (CLDN, ZO-1), and reduced crypt hyperplasia. It also suppresses inflammation (lower IL-1β, IL-6) and oxidative stress (↑GSH-Px, ↓MDA) through rebalancing gut microbiota, favoring beneficial Lactobacillus over pathogenic Clostridium. By attenuating apoptosis (Bak, BAX) and normalizing immune defenses, Aln-Gln can preserve mucosal continuity without antibiotics. These multifunctional capabilities of Aln-Gln make it a sustainable, economically viable alternative for combating antimicrobial resistance and enhancing poultry health in industrial production systems.

## Figures and Tables

**Table 1:** Effect of Aln-Gln on growth performance of *E. coli*-Challenged growing hens.

Period	Treatments					SEM	*p*-Value
	NC	C	G1	G2	G3		
**BWG** (g/bird/day)							
42	437.54	441.61	437.97	434.08	438.56	1.241	0.621
49	600.34 ^a^	515.91 ^c^	511.32 ^c^	560.41 ^b^	559.61 ^b^	3.213	0.040
56	707.04 ^a^	601.41 ^b^	614.58 ^a^	668.07 ^a^	671.71 ^a^	3.227	0.033
**ADFI** (g/bird/day)							
42	27.11	27.27	27.31	27.43	27.01	1.310	0.074
49	47.74 ^a^	29.81 ^c^	30.04 ^c^	40.19 ^b^	40.91 ^b^	1.246	0.016
56	54.21 ^a^	37.08 ^b^	38.01 ^b^	51.43 ^a^	52.00 ^a^	0.910	0.030
**FCE** (g/g)							
49	0.487 ^b^	0.356 ^a^	0.349 ^a^	0.449 ^b^	0.423	0.112	0.021
56	0.281 ^c^	0.329 ^b^	0.388 ^a^	0.299 ^c^	0.308	0.081	0.001

NC = Negative control group (no *E. coli* and no Aln-Gln); C = control group (*E. coli*-Challenged and no Aln-Gln); G1 = *E. coli*-Challenged and 0.1% Aln-Gln; G2 = *E. coli*-Challenged and 0.2% Aln-Gln; G3 = *E. coli*-Challenged and 0.3% Aln-Gln; superscripts in the same row indicate significant differences at *p* < 0.05; SEM = standard error of means; BWG, body weight gain in grams per bird per day; ADFI, average daily feed intake in grams per bird per day; FCE, feed conversion efficiency.

**Table 2:** The effects of Aln-Gln on the ileum morphology of *E. coli*-Challenged growing hens.

Items	Treatments					SEM	*p*-Value
	NC	C	G1	G2	G3		
Villus length, µm	844.33 ^a^	702.82 ^c^	780.11 ^b^	835.31 ^a^	831.42 ^a^	27.813	0.014
Crypt depth, µm	131.10 ^b^	155.62 ^a^	140.01 ^b^	134.02 ^b^	129.61 ^b^	7.610	0.033
V/C ratio	6.45 ^a^	4.52 ^c^	5.57 ^b^	6.23 ^a^	6.41 ^a^	0.369	0.001

NC = Negative control group (no *E. coli* and no Aln-Gln); C = control group (*E. coli*-Challenged and no Aln-Gln); G1 = *E. coli*-Challenged and 0.1% Aln-Gln; G2 = *E. coli*-Challenged and 0.2% Aln-Gln; G3 = *E. coli*-Challenged and 0.3% Aln-Gln; superscripts in the same row indicate significant differences at *p* < 0.05; SEM = standard error of means; V/C = villus length (µm)/crypt depth (µm).

**Table 3:** The effects of Aln-Gln on the serum cytokines of *E. coli*-Challenged growing hens.

Items	Treatments					SEM	*p*-Value
	NC	C	G1	G2	G3		
TNF-α, pg/mL	31.81 ^c^	52.33 ^a^	41.11 ^b^	40.83 ^b^	40.01 ^b^	2.671	0.017
IL-1β, pg/mL	344.22 ^b^	510.00 ^a^	490.54 ^a^	360.53 ^b^	340.30 ^b^	20.951	0.000
IL-6, pg/mL	14.47 ^b^	19.07 ^a^	17.99 ^a^	14.60 ^b^	14.51 ^b^	1.012	0.041
IL-4, pg/mL	119.54 ^a^	80.42 ^b^	71.40 ^b^	117.21 ^a^	120.83 ^a^	6.612	0.001
IL-10, pg/mL	65.76 ^a^	40.81 ^b^	38.22 ^b^	63.56 ^a^	64.00 ^a^	3.008	0.033

NC = Negative control group (no *E. coli* and no Aln-Gln); C = control group (*E. coli*-Challenged and no Aln-Gln); G1 = *E. coli*-Challenged and 0.1% Aln-Gln; G2 = *E. coli*-Challenged and 0.2% Aln-Gln; G3 = *E. coli*-Challenged and 0.3% Aln-Gln; superscripts in the same row indicate significant differences at *p* < 0.05; SEM = standard error of means.

**Table 4:** The effects of Aln-Gln on serum antioxidant status of *E. coli*-Challenged growing hens.

Items	Treatments					SEM	*p*-Value
	NC	C	G1	G2	G3		
GSH-Px, U/mL	124.56 ^a^	88.1 ^c^	99.00 ^b^	111.02 ^a^	115.91 ^a^	5.936	0.001
CAT, U/mL	69.01 ^a^	43.27 ^b^	47.91 ^b^	60.15 ^a^	61.72 ^a^	3.013	0.019
SOD, U/mL	370.66 ^a^	231.3 ^c^	280.52 ^c^	310.47 ^b^	315.55 ^b^	15.011	0.034
MDA, nomol/mL	7.02 ^c^	9.21 ^a^	8.71 ^a^	7.55 ^b^	7.39 ^b^	0.316	0.010

NC = Negative control group (no *E. coli* & no Aln-Gln); C = control group (*E. coli*-Challenged & no Aln-Gln); G1 = *E. coli*-Challenged & 0.1% Aln-Gln; G2 = *E. coli*-Challenged & 0.2% Aln-Gln; G3 = *E. coli*-Challenged & 0.3% Aln-Gln; superscripts in the same row indicate significant differences at *p* < 0.05; SEM = standard error of means.

**Table 5:** Effect of Aln-Gln supplementation on ileal NO and NOS levels in intestinal mucosa.

Parameter	Treatments					SEM	*p*-Value
	NC	C	G1	G2	G3		
NO	0.324 ^b^	0.594 ^a^	0.483 ^a^	0.318 ^b^	0.330 ^b^	0.034	0.021
TNOS	0.176 ^b^	0.318 ^a^	0.288 ^a^	0.175 ^b^	0.178 ^b^	0.014	0.046
INOS	0.223 ^b^	0.341 ^a^	0.317 ^a^	0.211 ^b^	0.228 ^b^	0.011	0.001

NC = Negative control group (no *E. coli* and no Aln-Gln); C = control group (*E. coli*-Challenged and no Aln-Gln); G1 = *E. coli*-Challenged and 0.1% Aln-Gln; G2 = *E. coli*-Challenged and 0.2% Aln-Gln; G3 = *E. coli*-Challenged and 0.3% Aln-Gln; superscripts in the same row indicate significant differences at *p* < 0.05.

**Table 6:** Composition of experimental diets on dry matter basis.

Ingredients (%)	NC	C	Aln-Gln Powder		
			G1	G2	G3
Corn	66.10	66.10	66.00	66.00	65.9
Soybean meal	29.00	29.00	29.00	28.90	28.99
Wheat Bran	0.200	0.200	0.200	0.200	0.200
Aln-Gln Powder	-	-	0.1	0.2	0.3
Met	0.20	0.20	0.20	0.20	0.20
Lys	0.20	0.20	0.20	0.20	0.20
Salt	0.30	0.30	0.30	0.30	0.30
Limestone	1.30	1.30	1.30	1.30	1.30
CaHPO3	1.70	1.70	1.70	1.70	1.70
Premix ^1^	1.00	1.00	1.00	1.00	1.00
Total	100.00	100.00	100.00	100.00	100.00
ME (MJ/kg)	11.98	11.98	11.98	11.98	11.98
CP %	18.4	18.4	18.4	18.4	18.4
Lys Dig. %	1.126	1.126	1.126	1.126	1.126
Met Dig. %	0.47	0.47	0.47	0.47	0.47
Thr Dig. %	0.68	0.68	0.68	0.68	0.68
Trp Dig. %	0.20	0.20	0.20	0.20	0.20
Arg Dig. %	1.22	1.22	1.22	1.22	1.22
Ile Dig. %	0.74	0.74	0.74	0.74	0.74
Val Dig. %	0.84	0.84	0.84	0.84	0.84
CF %	2.78	2.78	2.78	2.78	2.78
Ca %	1.078	1.078	1.078	1.078	1.078
P Av. %	0.45	0.45	0.45	0.45	0.45
Na %	0.023	0.023	0.023	0.023	0.023
Cl %	0.041	0.041	0.041	0.041	0.041

^1^ Per kilogram of diet, the following are provided: 11,000 IU of vitamin A, 3000 IU of vitamin D3, 20 IU of vitamin E, 3 mg of vitamin K3 (as menadione), 0.02 mg of vitamin B12, 6.5 mg of riboflavin, 10 mg of calcium pantothenate, 40.1 mg of niacin, 0.2 mg of biotin, 2.2 mg of thiamine, 4.5 mg of pyridoxine, 1000 mg of choline, 125 mg of ethoxyquin (antioxidant), 66 mg of Mn (as manganese oxide), 70 mg of Zn (as zinc oxide), 80 mg of Fe (as ferrous sulfate), 10 mg of Cu (as copper sulfate), 0.3 mg of Na (as sodium selenite), 0.4 mg of I (as calcium iodate), 0.67 mg of iodized salt.

**Table 7:** Gene sequences in real-time PCR primers.

Gene	Name	Product Length	Primer
*BAX*	transmembrane BAX inhibitor motif containing 6	96	F: GATGTGCATCGCCATCAACC R: CGGGCATAGAGAGCACTGAG
*ZO-1*	tight junction	136	F: CCTGCCAGCCATCATTCTGA R: TTGGCTCATAGCGCTTGTCA
*CLDN*	Claudin 10	127	F: TGGGTTTCTTTGGCTCCGTT R: GAGCACAGCCCACACAGTAT
*Bak*	BCL2 antagonist/killer 1	109	F: ACCCGGAGATCATGGAGA R: GATGCCTTGCTGGTAGACG
*NOS2*	nitric oxide synthase 2	119	F: CAGCACGTGGCTGAACAAAA R: TTTGGGAGCCGGAATCCATC
*AvBD1*	avian beta-defensin	100	F: GGATGCACGCTGTTCTTGGT R: TCCGCATGGTTTACGTCTGTC
*β-actin*	actin, beta	144	F: CTACACACGGACACTTCAAG R: ACAAACATGGGGGCATCAG

## Data Availability

Upon reasonable request, the datasets of this study can be made available from the corresponding author.
